# Integrating Bioinformatics and Experimental Validation to Identify Mitochondrial Permeability Transition-Driven Necrosis-Related lncRNAs that can Serve as Prognostic Biomarkers and Therapeutic Targets in Endometrial Carcinoma

**DOI:** 10.1007/s43032-024-01693-7

**Published:** 2024-10-01

**Authors:** Ting Zhou, Haojia Li, Qi Zhang, Shuangshuang Cheng, Qian Zhang, Yuwei Yao, Kejun Dong, Zheng Xu, Wan Shu, Jun Zhang, Hongbo Wang

**Affiliations:** 1 Department of Obstetrics and Gynecology, Union Hospital, Tongji Medical College, Huazhong University of Science and Technology, Wuhan, 430022 Hubei China; 2 Department of General Surgery, The Second Affiliated Hospital of Nanchang University, Nanchang, 330006 Jiangxi Province China; 3 Department of Obstetrics and Gynecology, Union Hospital, Tongji Medical College, Huazhong University of Science and Technology, Wuhan, 430022 Hubei China; 4 Clinical Research Center of Cancer Immunotherapy, Wuhan, 430022 Hubei China

**Keywords:** Endometrial carcinoma, Prognostic signature, LncRNAs, Mitochondrial permeability transition, Biomarkers

## Abstract

Endometrial carcinoma (EC) is a common malignant tumor in women with high mortality and relapse rates. Mitochondrial permeability transition (MPT)-driven necrosis is a novel form of programmed cell death. The MPT-driven necrosis related lncRNAs (MRLs) involved in EC development remain unclear. We aimed to predict the outcomes of patients with EC by constructing a novel prognostic model based on MRLs and explore potential molecular functions. A risk prognostic model was developed utilizing multi-Cox regression in conjunction with the Least Absolute Shrinkage and Selection Operator (LASSO) regression algorithm, which was based on MRLs. The predictive efficacy of the model was evaluated through receiver operating characteristic (ROC) curve analysis, as well as nomogram and concordance index (C-index) assessments. Patients were categorized into high- and low-risk groups based on their median risk scores. Notably, the high-risk group exhibited significantly poorer overall survival (OS) outcomes. Gene ontology (GO) and Gene set enrichment analysis (GSEA) demonstrated that Hedgehog and cell cycle pathways were enriched in the high-risk group. Tumor Immune Dysfunction and Exclusion (TIDE) displayed that patients in the high-risk group showed a high likelihood of immune evasion and less effective immunotherapy. A significant disparity in immune function was also observed between two groups. Based on the nine-MRLs, drug sensitivity analysis identified several anticancer drugs with potential efficacy in prognosis. Meanwhile, the results demonstrated that OGFRP1 plays a carcinogenic role by affecting mitochondrial membrane permeability in EC. Therefore, the risk model constructed by nine MRLs could be used to predict the clinical outcomes and therapeutic responses in patients with EC effectively.

**Supplementary Information:**

The online version contains supplementary material available at 10.1007/s43032-024-01693-7.

## Introduction

Endometrial carcinoma (EC) is one of the most popular cancers in women, with increasing incidence and disease-associated mortality worldwide [1]. According to Global Cancer Statistics 2020, 417,000 new cases and 97,000 EC deaths occurred in 2020 worldwide [2]. More than 100,000 new cases are anticipated by 2040 [3]. Currently, multiple treatment methods, such as surgery, chemotherapy, brachytherapy, and radiotherapy, are used to treat EC [4]. Although 67% of EC patients present with early-stage disease, which is associated with an 81% 5-year OS, the 5-year OS of stage IVA and IVB EC are just 17% and 15%, respectively [5]. With recurrence, the survival rate sharply declines, even in patients with early-stage EC, with the 5-year OS dropping to 55% for pelvic recurrence and 17% for extra-pelvic recurrence [6]. Therefore, new methods to improve the prognosis of patients with EC are needed.

Mitochondria are an important component of respiration and pivotal regulators of metabolism, involved in various cellular processes. When the transient passage of low molecular weight solutes across the inner mitochondrial membrane is abruptly induced, subsequent disruption and swelling of the mitochondria can result [7]. This mitochondrial permeability transition (MPT) can then drive necrosis, a distinct form of regulated cell death that is induced by intracellular microenvironmental perturbations, including severe cytosolic Ca2 + overload and oxidative stress [8]. Its association with severe diseases and involvement in numerous cellular states allow it to be classified as a necrotic morphotype [9]. Considering the correlation between MPT-induced necrosis and carcinoma, investigating this mechanism in EC could enable the development of efficacious therapeutic approaches.

Long non-coding RNAs (lncRNAs) are greater than 200 nucleotides in length, function as regulators of gene expression at either the post-transcriptional or transcriptional level but cannot be translated into proteins [10]. Owing to their versatile nature, lncRNAs exhibit behavior comparable to that of oncogenes or tumor suppressors. They govern a variety of cellular processes linked to cancer, encompassing cell proliferation, metastasis, and stemness [11]. As previously reported, downregulation of HOTAIR expression substantially decreases the colony count and proliferation of HEC-1A cells. Additionally, downregulation of HOTAIR expression induced G0 /G1 phase cell cycle arrest [12]. The cancer-specific homologous expression of lncRNAs in mammals provides evidence that lncRNAs can serve as therapeutic targets and biomarkers [13].

In this study, the relationships between MPT-driven necrosis related lncRNAs (MRLs) and EC is being investigated. We identified specific biomarkers associated with EC prognosis using comprehensive bioinformatics analyses, providing a new perspective on EC prognosis. We constructed a risk score prognostic model for nine MRLs in EC patients by univariate cox, lasso, and multivariate cox regression analysis. The risk value for each patient was determined using a risk scoring model formula, which subsequently allowed for the categorization of patients into low-risk and high-risk groups based on the median value. A comprehensive set of correlation and prognostic analyses was conducted within these groups to evaluate the clinical significance and prognostic utility of the proposed model. Additionally, the role of OGFRP1 in endometrial cancer (EC) was partially validated.

## Results

### Constructing a Risk Prediction Model of MRLs

**Fig. 1:**
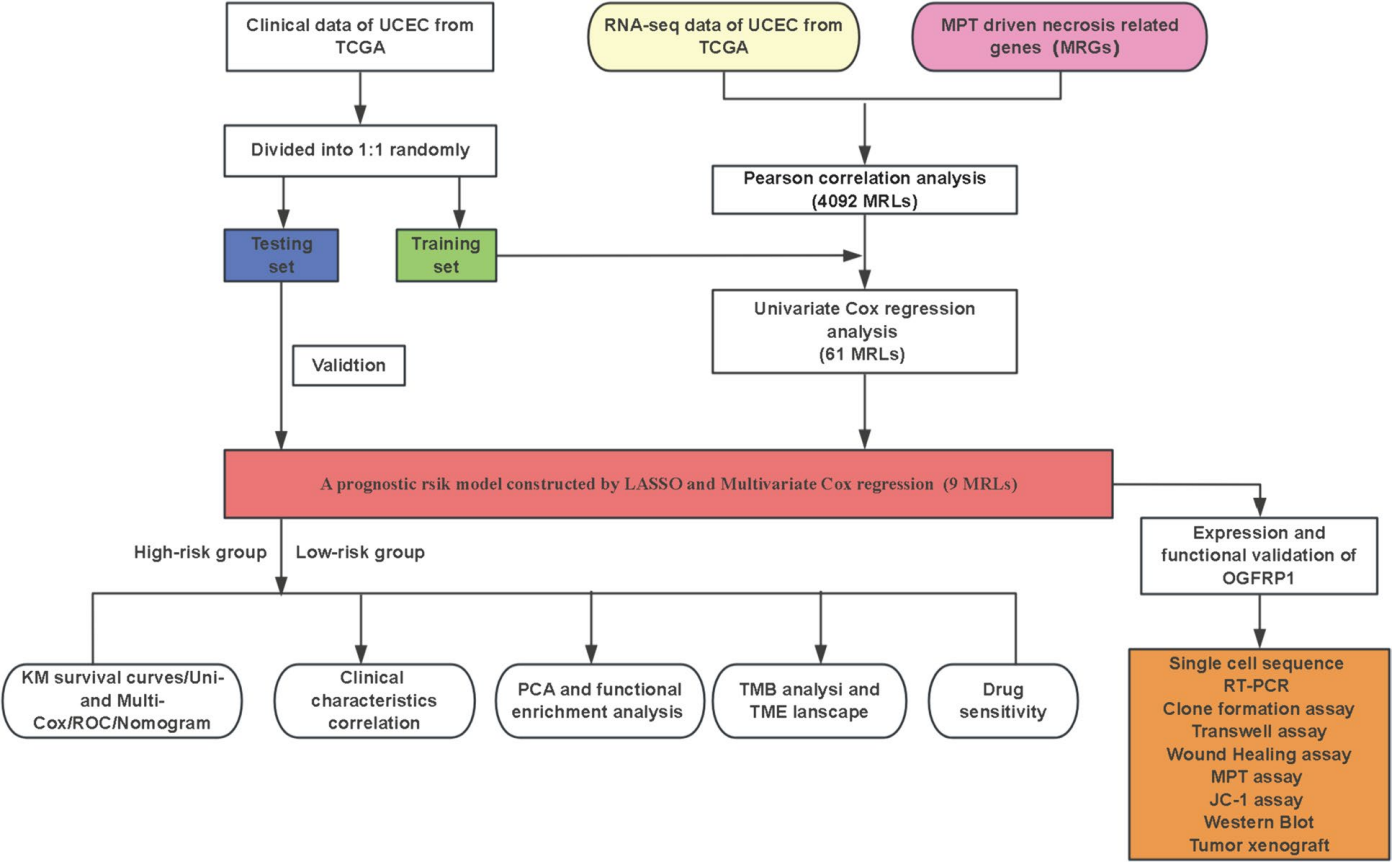
The flow chat of study

**Fig. 2:**
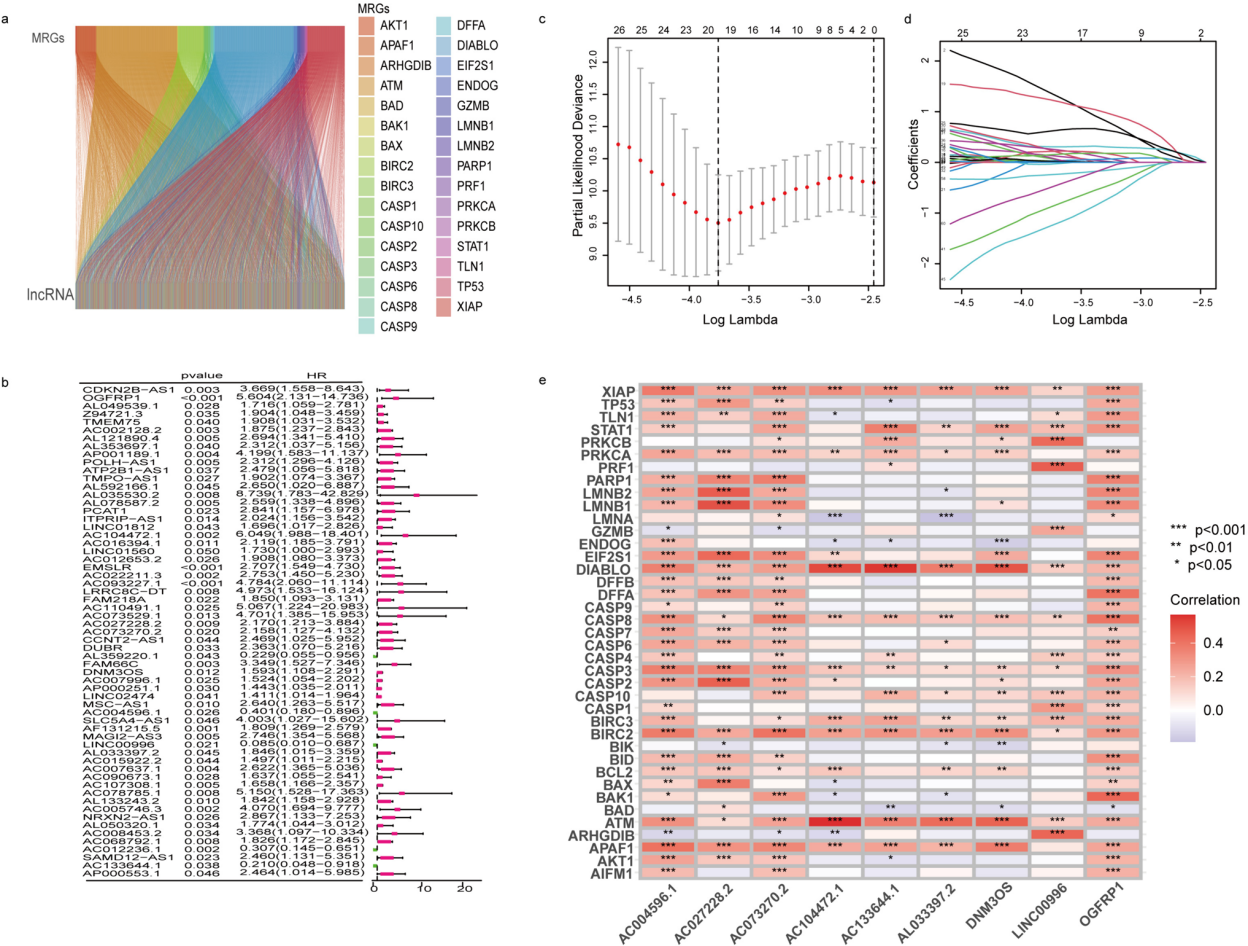
Construction of the risk model with nine MRLs. **a**. Visualizing the relationship between MRGs and MRLs using a Sankey diagram. **b**. Displaying MRLs pertinent to prognostic in EC. **c**. Analysis of LASSO coefficients pertaining to MRLs. **d**. Cross validation of MRLs using LASSO. **e**. Examining the relationship between MRGs and MRLs

Figure 1 depicts the workflow of this study. First, 16,877 lncRNAs were discovered in the RNA-seq data obtained from patients with EC, using the most recent lncRNA annotation file (see Supplementary S1). These lncRNAs were assessed for Pearson’s correlation with 31 MPT-driven necrosis-related genes (MRGs) to yield 4092 MRLs (**| HR |**> 0.4, *p* < 0.001). A Sankey chart displays the visualized results of the co-expression relationship between lncRNAs and MRGs (Fig. 2a).

A total of 368 patients were categorized randomly into two groups: the Training group (*n* = 184) and the Testing group (*n* = 184). The validation results of the clinical grouping confirmed its appropriateness, revealing no significant disparities in various clinical factors between the two groups. Subsequently, the training cohort was used to develop an ideal model to predict the prognosis of EC, while both the testing cohort and the entire patient cohort were employed for evaluation purposes. First, univariate Cox regression analysis identified 61 MRLs that associated with the EC patient’s outcomes were then included in our study (*p* < 0.05, Fig. 2b). Least absolute shrinkage and selection operator (LASSO) and Multivariate Cox regression analysis screened nine potential MRLs to construct a risk prognostic model in EC patients (Fig. 2c-e). The risk model was constructed using nine MRLs, including OGFRP1, AC104472.1, AC027228.2, AC073270.2, DNM3OS, AC004596.1, LINC00996, AL033397.2, and AC133644.1. We calculated the risk score by employing the multivariate Cox regression formula: Risk score = (5.77115520944495 × OGFRP1) + (2.68037998172689 × AC104472.1) + (2.27810203936939 × AC027228.2) + (0.846611717086348 × AC073270.2) + (0.967693641700537 × DNM3OS) + (-3.24302378158702 × AC004596.1) + (-3.91637555748088 × LINC00996) + (0.866174277178801 × AL033397.2) + (-2.18072658211276 × AC133644.1). The correlation between the nine MRLs and MRGs were shown in Fig. 2e.

**Fig. 3:**
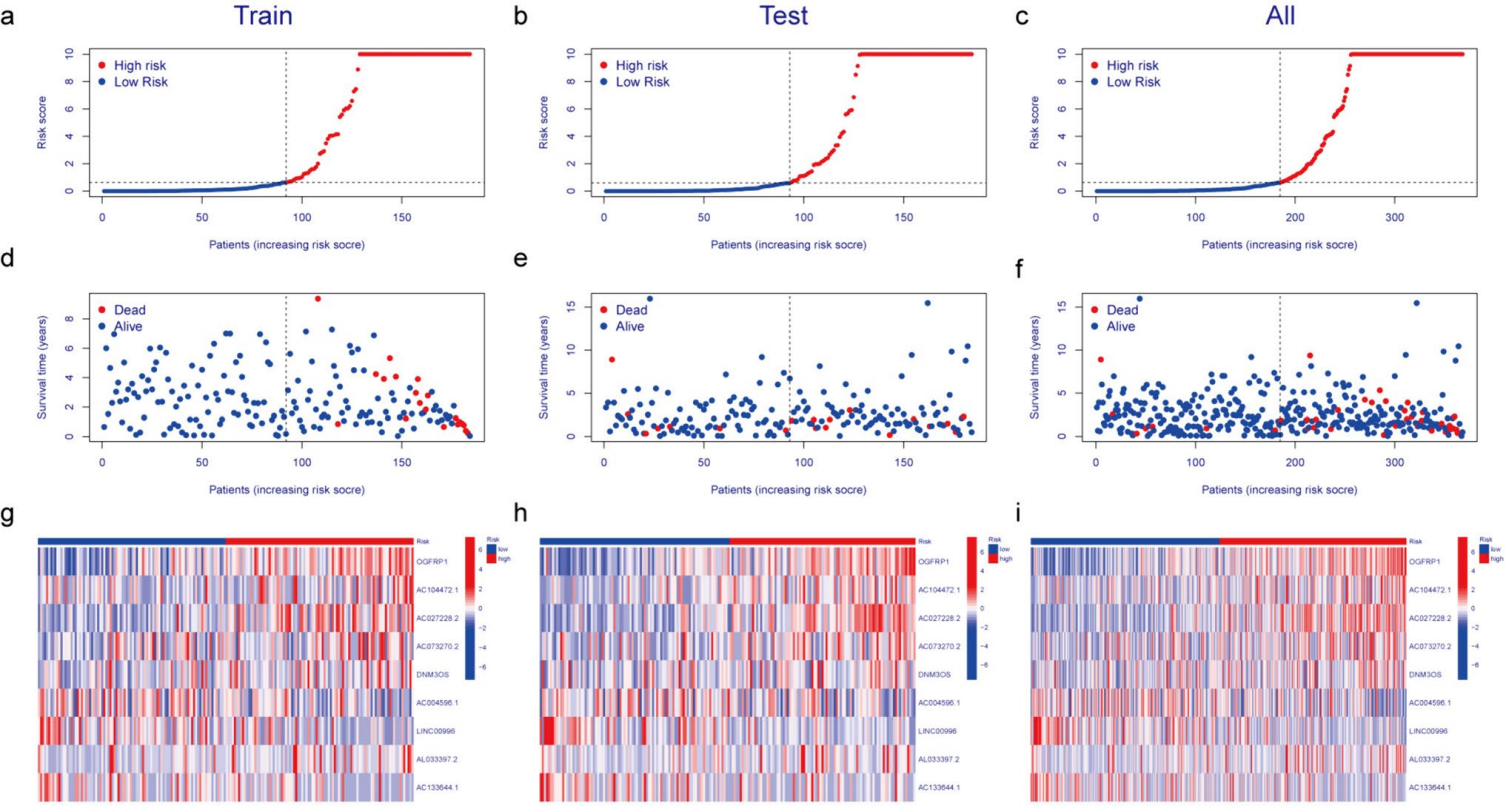
Development and validation of the prognostic MRLs signature. **a**-**c**. The distribution of patients based on escalating risk scores. **d**-**f**. The correlation between risk scores and survival outcomes. **g**-**i**. Heatmaps display levels of nine MRLs in two groups

The risk value for each patient was determined using the risk scoring model formula, subsequently classifying patients into low-risk and high-risk groups based on the median value. In the training cohort, those categorized in the high-risk group exhibited notably higher risk scores in contrast to their low-risk counterparts (Fig. 3a). And as the risk score increased, mortalities of patients also gradually increased (Fig. 3d). The heatmap showed that OGFRP1, AC104472.1, AC027228.2, AC073270.2, DNM3OS, and AL033397.2 exhibited an upward expression trend in the high-risk group contrary to AC004596.1, LINC00996, and AC133644.1 (Fig. 3g). To further validate the previously mentioned results, identical analyses were conducted on two additional subgroups. Consistent findings were also confirmed in the testing cohort (Fig. 3b, e, and h) as well as in the cohort comprising all patients (Fig. 3c, f and i).

### Predicting Outcomes in High- and Low-Risk Patients Based on Clinical Features

**Fig. 4:**
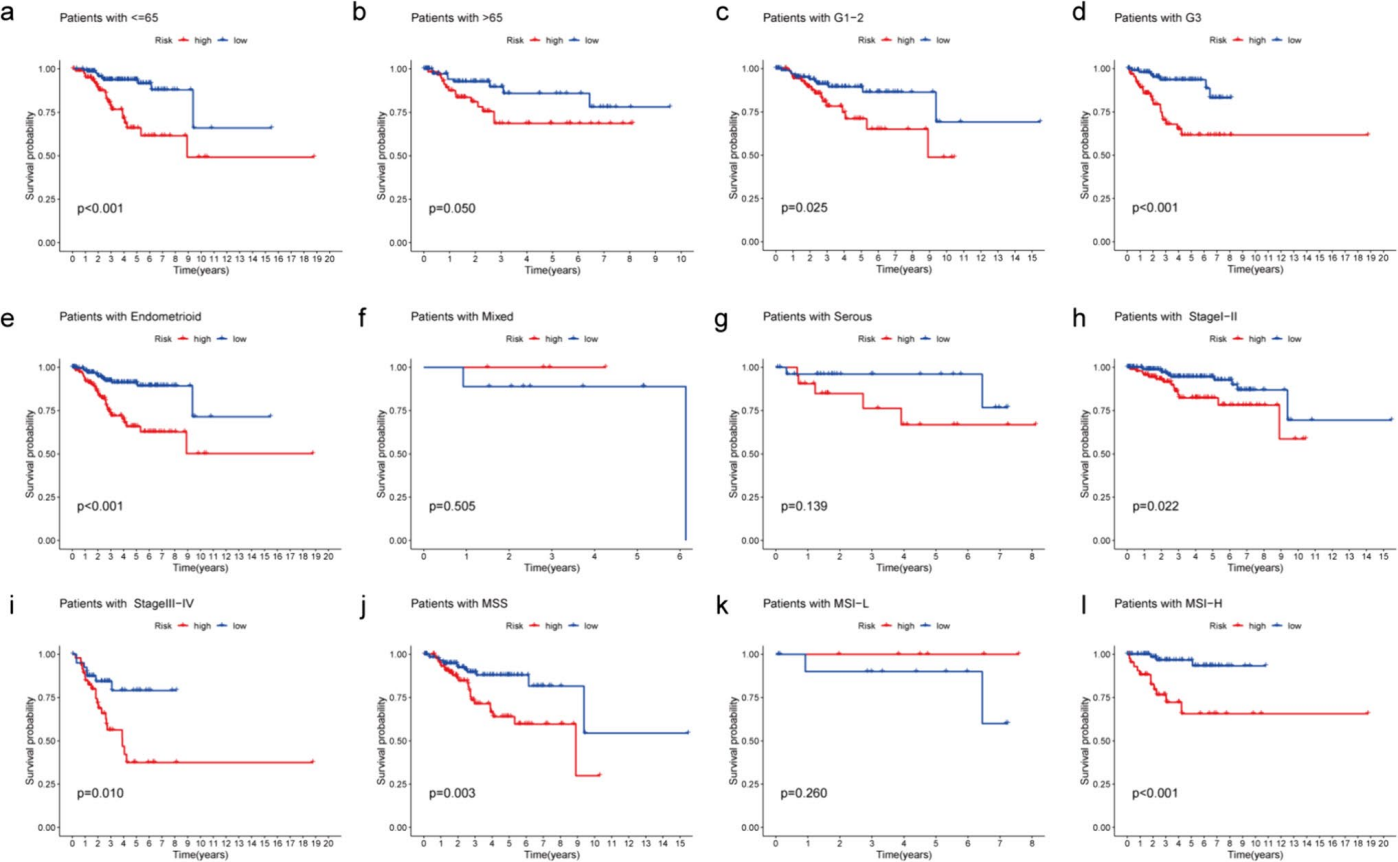
KM survival curves of risk subgroups among patients stratified by various clinicopathological characteristics. Age (**a**, **b**), Grade (**c**, **d**), Histology (**e**, **f**, **g**), Stage (**h**, **i**) and MSI (**j**, **k**, **l**)

To determine whether the risk score can effectively forecast patient mortality, we analyzed the differences in OS between the low-risk group and the high-risk group in TCGA according to clinicopathological characteristics, such as age, stage, grade, histology, and microsatellite instability (MSI). The KM survival curves showed that for most clinical variables, compared with patients with low-risk scores, high-risk score patients had a poor prognosis, except for those with MSI-L and mixed histology (Fig. 4). Collectively, these findings clearly indicated that the nine MRLs we investigated is applicable to a wide range of clinical variables and has remarkable potential prognostic value in EC.

### Validation of the Model Accuracy and Construction of the Nomogram

**Fig. 5:**
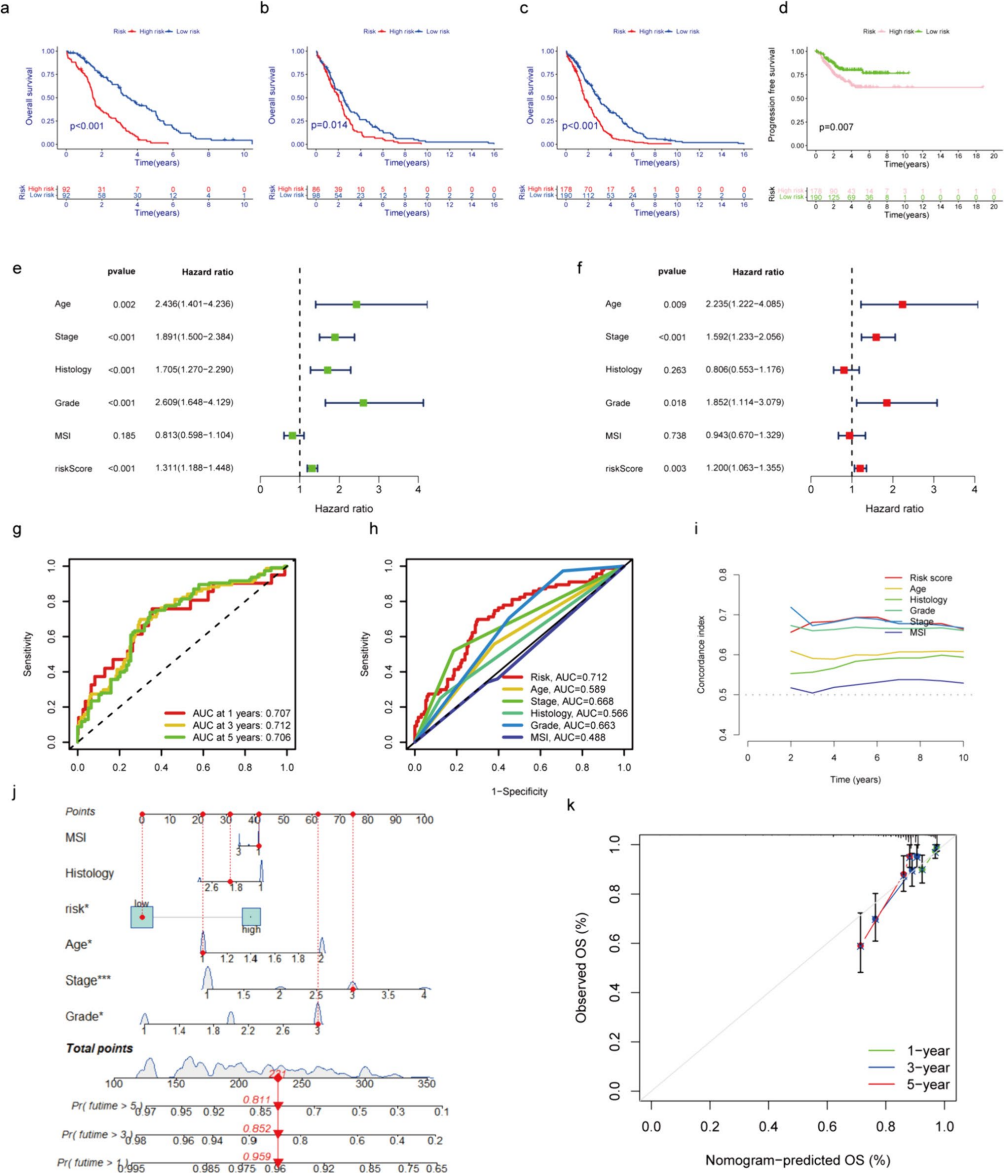
The ability of clinical predict and independent prognostic of nine MRLs in EC. **a**-**d**. Kaplan–Meier (KM) survival curves exhibit OS and PFS of EC patients in the high- and low-risk categories (Train, Test, and All). **e**. Univariate Cox regression showing clinical variables and signature MRLs. **f**. Multivariate Cox regression analysis incorporating clinical variables and signature MRLs. **g**. Prediction of 1-,3-, and 5-year OS for EC patients in all set. **h**. Comparison between the predictive risk model and clinicopathological characteristics. **i**. C-index evaluating the risk model's performance. **j**. The combination of risk and clinicopathological features to forecast 1-,3-, and 5-year OS in EC patients. **k**. Calibration curves illustrating the risk model's accuracy in forecasting 1-,3-, and 5-year OS in EC patients

Based on the Kaplan–Meier survival curves, patients categorized in the high-risk group exhibited significantly lower OS and progression-free survival (PFS) in comparison to those in the low-risk group. This observation indicates that the high-risk group is associated with a generally poorer prognosis (Fig. 5a–d). The risk score derived from the nine MRLs was identified as independent prognostic factor distinct from other clinicopathological features, as determined using both uni- and multi-Cox regression analysis (Fig. 5e–f). The prognostic prediction for patients, based on the AUC of the ROC curve derived from risk scores, yielded AUC values of 0.707, 0.712, and 0.706 for 1, 3, and 5 years, respectively (see Fig. 5g). When juxtaposed with other clinical characteristics in EC, the risk score exhibited the highest AUC, suggesting its commendable predictive capability (Fig. 5h).

To evaluate model precision, the 10 years C-index was computed using cross-validation and non-cross-validation methods, which showed a higher ranking compared to other clinical factors (Fig. 5i). This suggests that the risk score could serve as a dependable prognostic indicator for patients with EC, particularly in terms of long-term clinical outcomes. To enhance the utility of the risk model, a predictive nomogram was constructed by aggregating designated risk scores pertaining to pertinent clinical factors. This development facilitated the precise estimation of survival likelihood. Based on this nomogram, 1-, 3- and 5-year OS probabilities of the enrolled patients were 0.959, 0.852, and 0.811, respectively (Fig. 5j). Strong agreement between predicted survival and clinical outcomes was demonstrated by calibration curves, which validated the nomogram predictions (Fig. 5k). In summary, these illustrated that the risk model constructed by nine MRLs may serve as reliable prognostic indicators for precisely predicting clinical outcomes in EC.

**Fig. 6:**
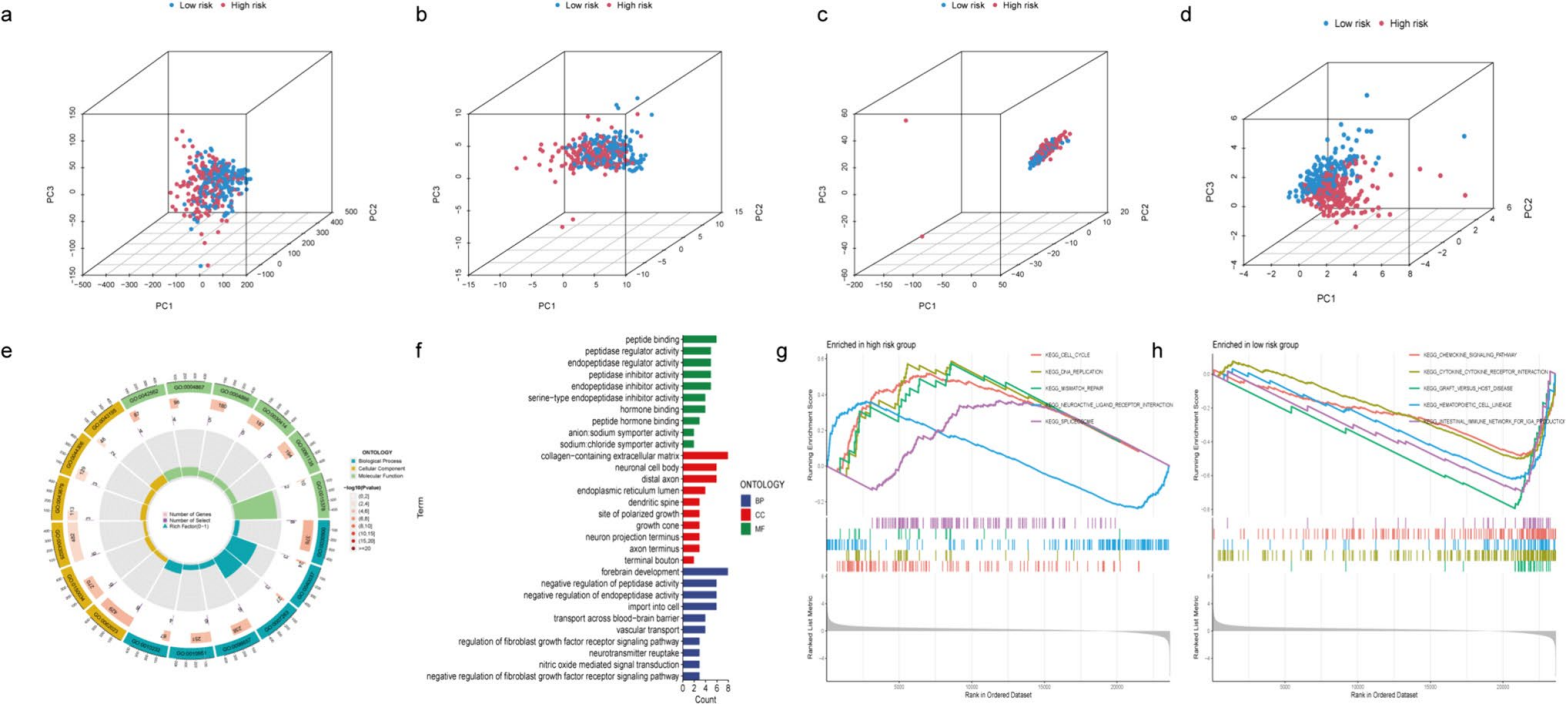
The results of PCA, GO and GSEA analyses. **a**. Plot for all genes. **b**-**c**. MPT driven necrosis-related genes and lncRNAs, respectively. **d**. The lncRNAs at risk. **e**–**f**. GO analysis highlighting the diversity of MFs, BPs and CCs. **g**-**h**. GSEA shows the top five enriched pathways in both high- and low-risk groups

Building upon the previous section of the study, the concepts of accuracy and specificity have been thoroughly examined. To further investigate the model's functionality, PCA was employed to demonstrate the distinct distributions between the high- and low-risk datasets. No noticeable variations in the patterns of expression for whole genes, MRGs, or MRLs between the two risk datasets (Fig. 6a–c). Only the risk-related lncRNAs demonstrated the most robust ability to differentiate among patients in the high- and low-risk categories (Fig. 6d).

### Biological Functional GO Analysis and GSEA

Subsequently, 219 differentially expressed genes (DEGs) were identified through the comparison of mean expression levels between the low-risk and high-risk categories (*P* < 0.05, log_2_ FC > 1, see Supplementary S2). To investigate the biological attributes of DEGs, GO analysis was performed. molecular biological processes (BPs) demonstrates that DEGs dramatically contribute to "vascular transport" and "regulation of the fibroblast growth factor receptor signaling pathway." A substantial abundance of "collagen-containing extracellular matrix" was observed in the cellular components (CCs) domain. In contrast, DEGs that were enriched for molecular functions (MFs) exhibited significant associations with the terms "extracellular matrix structural constituent" and "cytokine binding" (see Fig. 6e–f and Supplementary S3). DEGs were primarily implicated in the reprogramming of the extracellular matrix, which contributes to metastasis and drug resistance in numerous types of cancer. Moreover, immune-related and tumor development-associated enrichment pathways, including "cell cycle," "hedgehog signaling pathway," and "mismatch repair" were activated in the high-risk group (see Fig. 6g and Supplementary S4). Furthermore, the low-risk group exhibited a substantial increase in the expression of "chemokine signaling pathway" and "cytokine-cytokine receptor interaction" genes (Fig. 6 h). NES were showed in Supplementary S4.

### Mutational Landscape in the High‑ and Low‑risk Groups

**Fig. 7:**
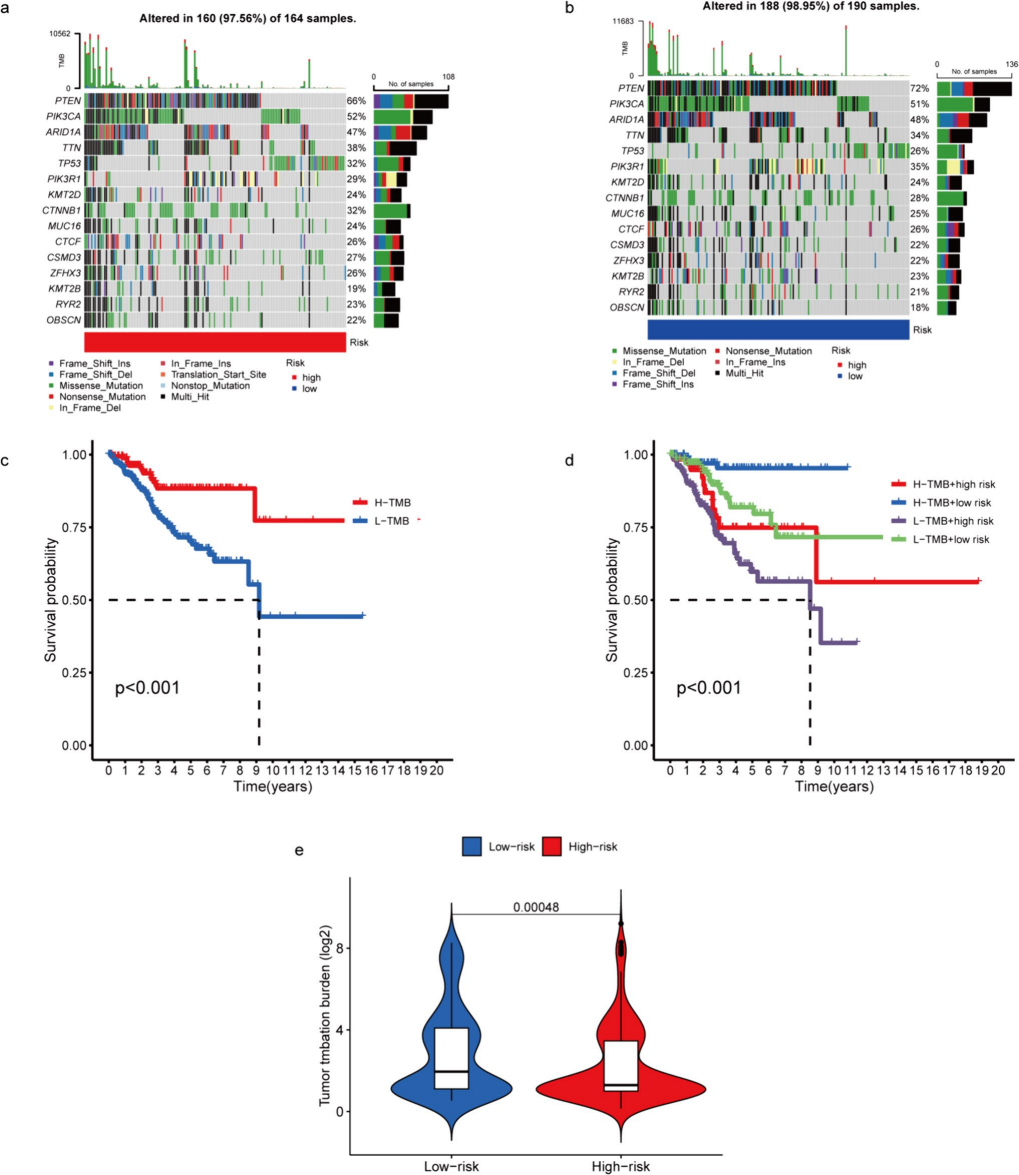
The distinction of TMB patterns between high- and low-risk groups. **a**-**b**. The waterfall plot displayed the key mutation genes in the high-risk and low-risk groups. **c**-**d**. Survival graphs for the high and low TMB groups in EC alongside a merged TMB-risk survival graph. **e**. Differences in TMB between the high- and low-risk groups

We obtained data on somatic mutations from TCGA to compare the evolution of somatic mutations between patients with high- and low-risk scores. The following genes exhibited the highest frequencies of mutations: PTEN, PIK3CA, ARID1A, TTN, TP53, PI3KR1, KMT2D, CTNNB1, MUC16, CTCF, CSMD3, ZFHX3, KMT3B, RYR2, OBSCN. The prevalence of mutations in these genes was higher in the low-risk than in the high-risk group (Fig. 7a–b). And the result of violin chart also showed a significant difference in TMB between the high- and low-risk groups (Fig. 7e). TMB was computed based on these mutated genes, and further investigation was conducted on the disparity in survival rates between patients with high and low TMBs. As shown in Fig. 7c, Patients with high TMB had a favourable OS compared to patients with low TMB. Patients with high TMB and low-risk score showed the highest likelihood of survival, which could be a supplement to predict patient prognosis using TMB alone (Fig. 7d).

### Analyses of Tumor Microenvironment (TME) Landscape and TIDE

**Fig. 8:**
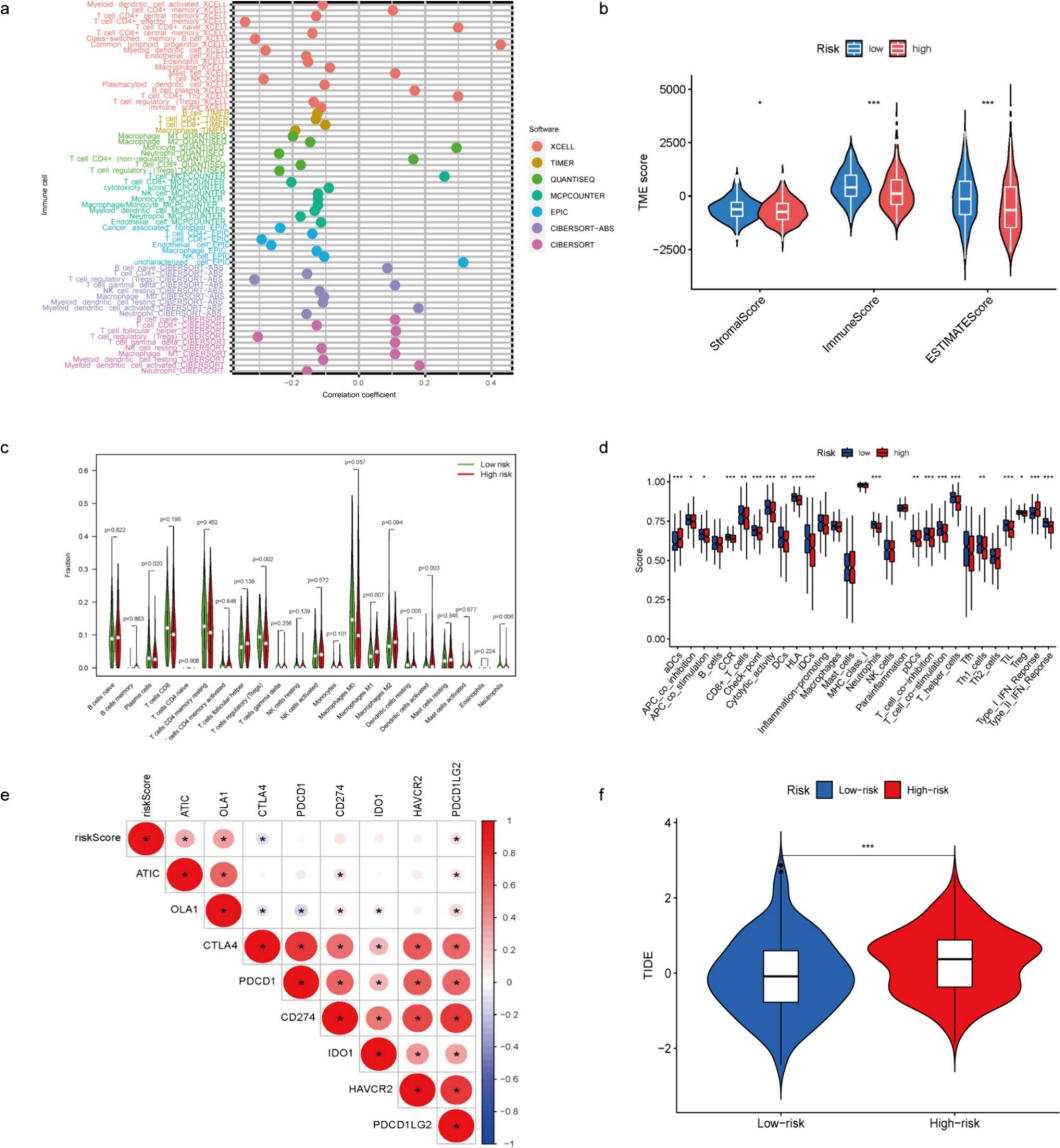
Alteration of TME between patients with high- and low-risk scores. **a**. Generating heatmaps to evaluate immune responses using various tools like XCELL, TIMER, QUANTISEQ, MCPCOUNTER, EPIC and CIBERSORT. **b**. Assessing discrepancies in immune microenvironment scores between high- and low-risk group. **c**. Illustrating the distribution of tumor-infiltrating immune cells through a violin plot for both high- and low-risk groups. **d**. Contrasting the enrichment scores of immune-related pathways in high-risk versus low-risk groups. **e**. Investigating the correlation between immune checkpoints and risk scores. **f**. Analysing TIDE between the high- and low-risk groups

Given the established correlation between immune responses and EC progression, we evaluated the influence of the risk signature on immune responses within TME. Immune-related heatmaps generated by the MCP-COUNTER, TIMER, QUANTISEQ, CIBERSORT, XCELL and EPIC algorithms are showed in Fig. 8a, with a high overlap between the results of the different algorithms. The "ESTIMATE" algorithm was employed to compute immune-related scores. Obviously, the stromal, immune, and estimate scores were markedly lower in the high-risk group (Fig. 8b), which suggests a compromised immune response and elevated tumor purity. Consequently, the unfavorable prognosis observed in the high-risk cohort may, in part, be attributable to diminished immune functionality. Given the crucial importance of the TME, we utilized ssGSEA to assess the enrichment scores of 22 immune cell types and 29 immune-related pathways during the transition from low- to high-risk category. These findings indicated that many immune cell types were highly abundant in low-risk group, including T cell regulatory (Tregs), dendritic cells resting, dendritic cells activated, and neutrophils. Additionaly, the majority of immune-related pathways exhibited lower levels of enrichment in the high-risk group: CCR, CD8^+^ T cells, cytolytic activity, T cell co-inhibition, HLA, T cell co-stimulation, MHC class I, Tregs, Th1 cells, TIL, and type II IFN response. ADCs and type I IFN response showed significantly elevated levels in the high-risk group (Fig. 8c and d). High CD8^+^ cell numbers in EC are linked to favorable prognoses. Additional differences in the expression of immune checkpoints were identified between the two groups, thus highlighting the revolutionary impact of immune checkpoint inhibitors in the treatment of malignancies (Fig. 8e). Furthermore, the TIDE algorithm (http://tide.dfci.harvard.edu/) was employed to analyze variances in immunotherapy response among patients categorized into high- and low-risk groups. The high-risk group exhibited a higher TIDE score than the low-risk group (Fig. 8f). Hence, immunotherapy might produce a more significant therapeutic impact in low-risk patients than in high-risk patients owing to the decreased likelihood of immune evasion.

### Screening for Potential Drugs in EC

**Fig. 9:**
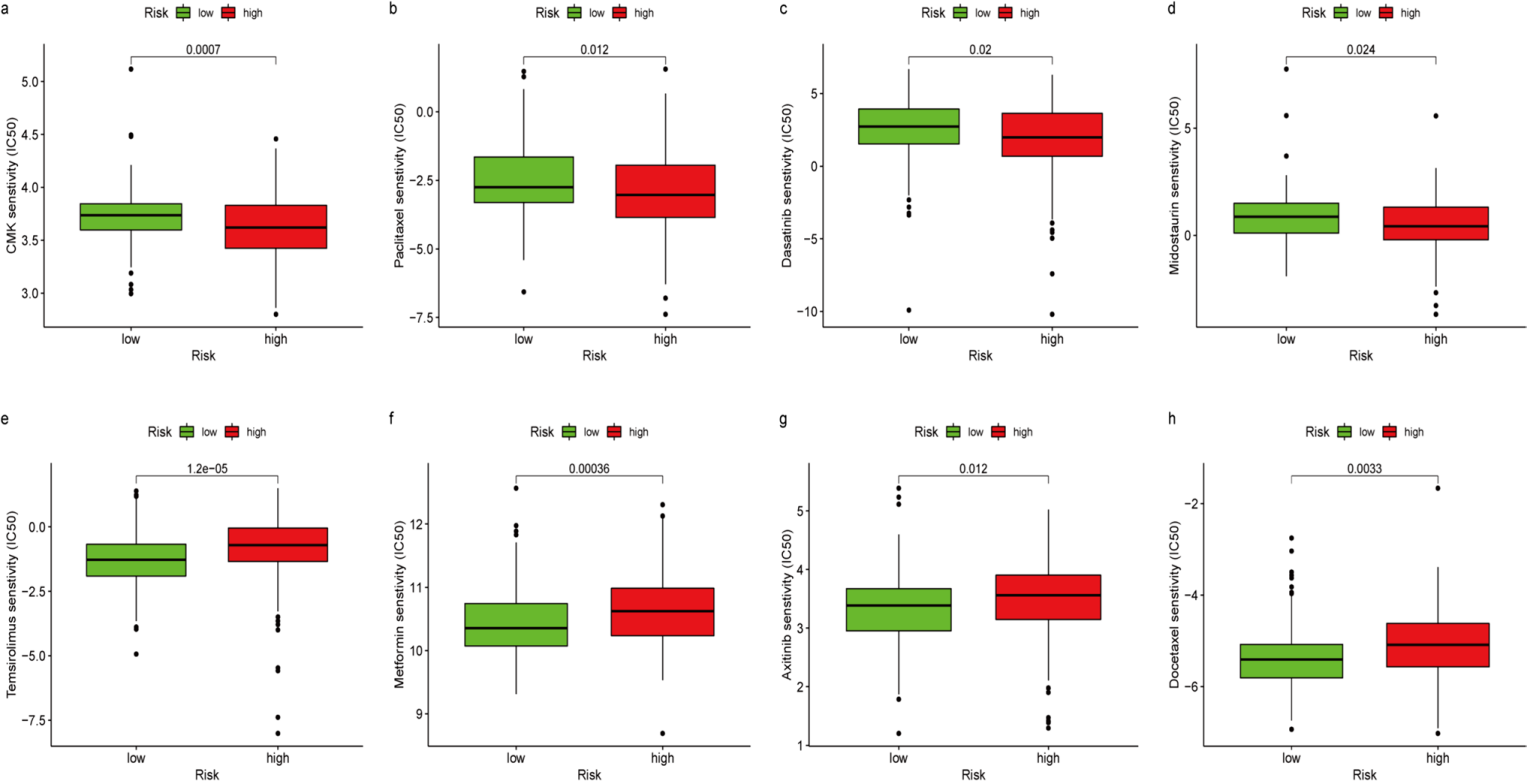
Identification of potential medications for treating EC involves analyzing the differences in IC50 values between high- and low-risk groups for eight drugs: CMK (**a**), Paclitaxel (**b**), Dasatinib (**c**), Midostaurin (**d**), Temsirolimus (**e**), Metformin (**f**), Axitinib (**g**) and Docetaxel (**h**)

Chemotherapy, along with immunotherapy, is a critical treatment option for patients diagnosed with EC. To validate the prospective therapeutic value of this risk model, we compared the IC50 values of the two groups for specified chemotherapy drugs. CMK, midostaurin, dasatinib and paclitaxel exhibited lower IC50 values when administered to high-risk patients (Fig. 9a–d). Conversely, when treating patients classified as low-risk, axitinib, docetaxel, temsirolimus and other drugs, like metformin, demonstrated reduced IC50 values and exhibited a positive correlation with risk scores (Fig. 9e–h). To guarantee consistent treatment procedures, these medications require care.

### Single Cell Sequencing Verified the Gene Features in Tumor Tissues

**Fig. 10:**
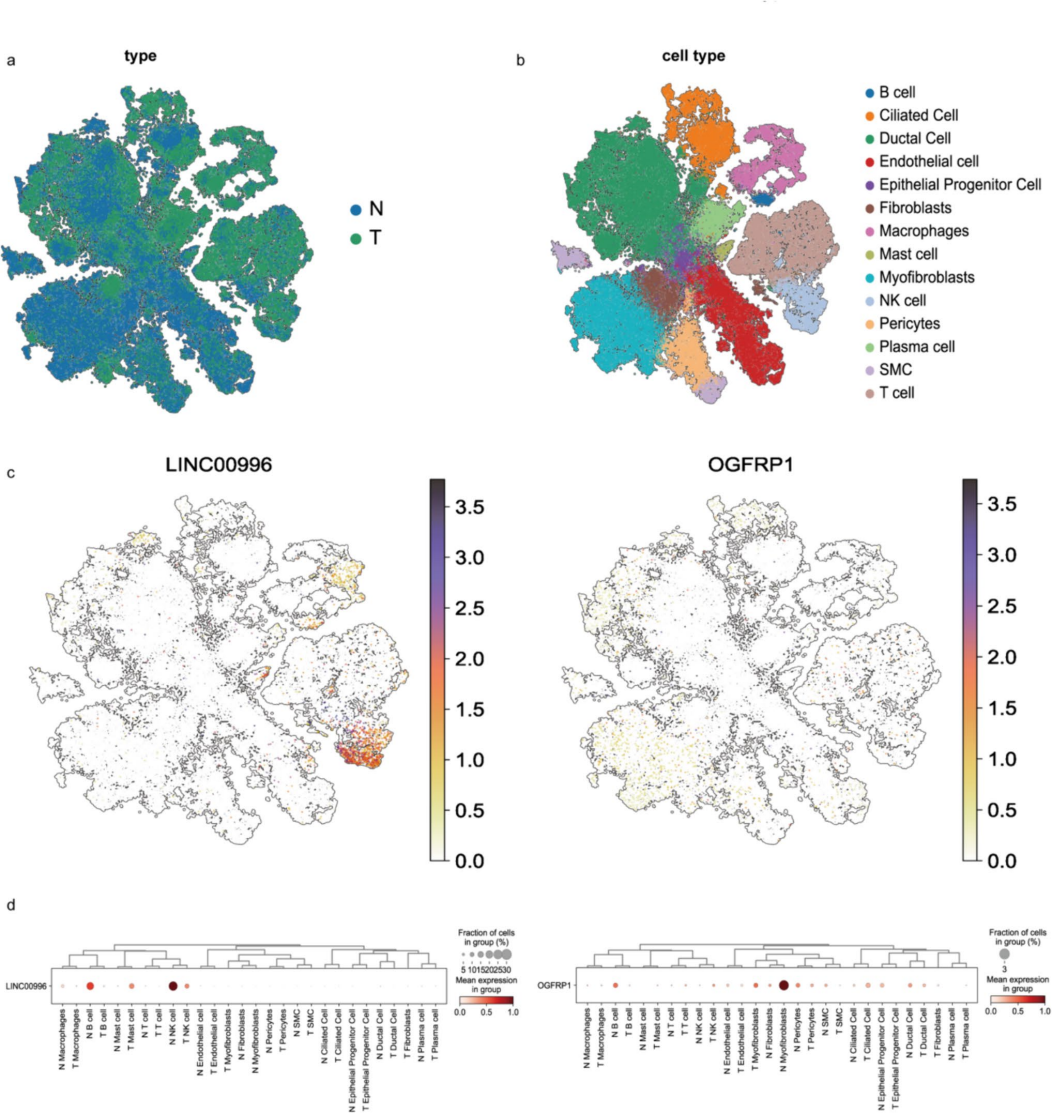
Single cell sequencing verified the gene features in EC. **a**-**b**. Gene degradation and cell subpopulation clustering. **c**-**d**. Visualization of differential expression of LINC00996 and OGFRP1 in different cell subpopulations

After removing cells of low quality, 99,215 endometrial cells were clustered into 14 clusters based on tagged genes that were traced back to their origin (see Supplementary S7). These clusters were formed by the subsequent processes of normalization, dimensionality reduction, and integration (Fig. 10a and b). Figure 10 c and d illustrated the variations in transcriptional levels of LINC00996 and OGFRP1 in the 14 clusters of cells. Notably, OGFRP1 exhibited high expression in mast, T, NK, ciliated and plasma cells of Tumor, while LINC00996 was prominently expressed in macrophage, NK and B cells of Normal.

### Validation of Expression of lncRNA OGFRP1 in EC

**Fig. 11:**
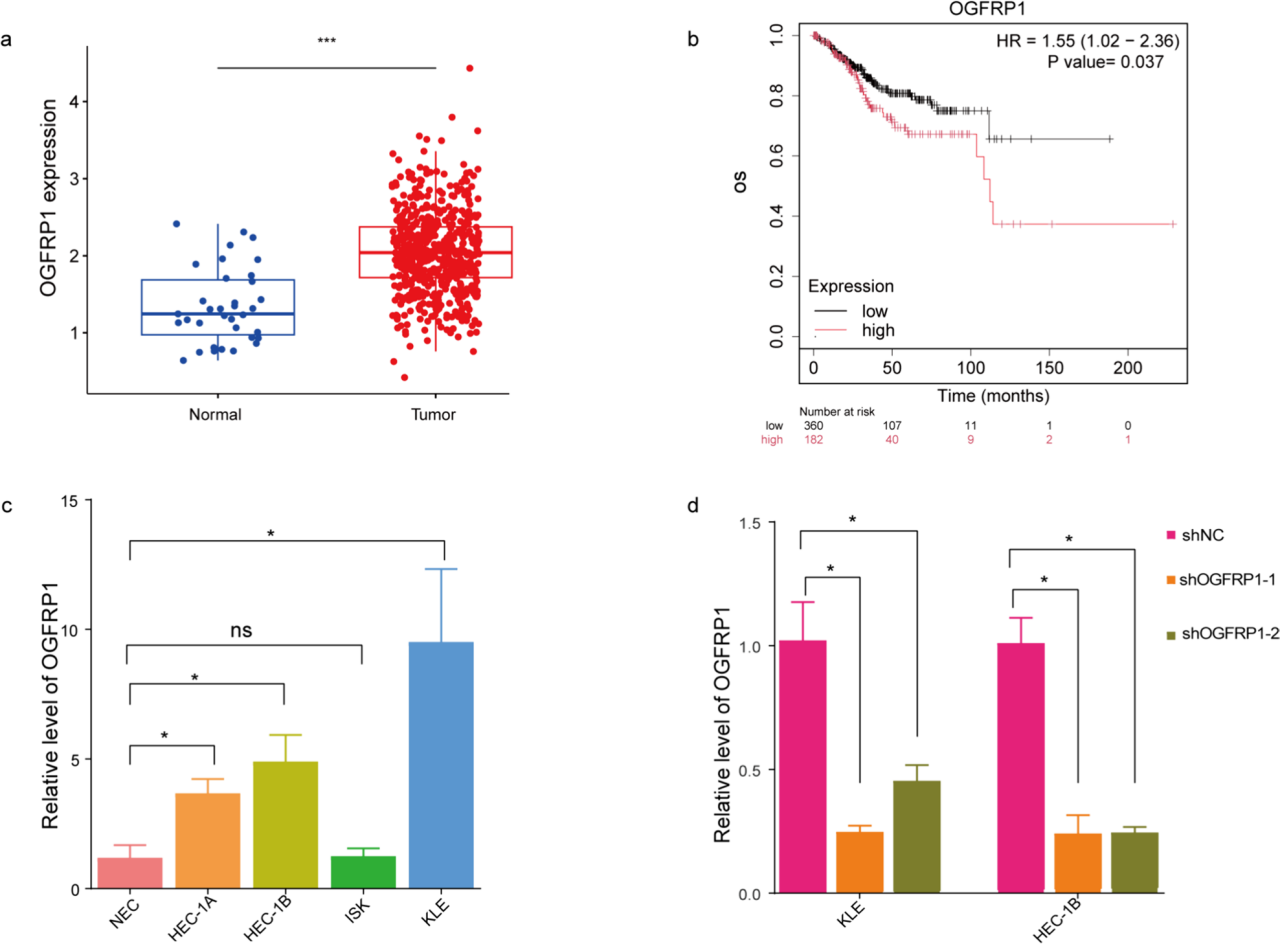
The expression of OGFRP1 in EC. **a**. The level of OGFRP1 expression in EC patients was obtained from TCGA. **b**. KM Plotter database exhibited the relationship between expression of OGFRP1and OS in EC patients. **c**. The OGFRP1 expression in different endometrial cancer cells and normal endometrial cells. **d**. Knockdown efficiency after transfection with plasmid shOGFRP1 in KLE and HEC-1B cells. *, *p* < 0.05; **, *p* < 0.01; ***, *p* < 0.001

In order to verify the prediction effect of this risk model, we choose lncRNA OGFRP1 for experimental validation. The external KM Plotter database revealed that OGFRP1 are significant predictive of poor prognosis, exhibiting a statistically significant correlation with OS (HR = 1.55 [1.02–2.36], *P* = 0.037, as shown in Fig. 11b), which corroborated our results. Notably, OGFRP1 exhibited higher expression in EC tissues than Normal, according to information from TCGA database (Fig. 11a). Meanwhile, OGFRP1 was also highly expressed in HEC-1A, HEC-1B, ISK, and KLE cells compared with Normal endometrial cells (Fig. 11c). HEC-1B and KLE cells were selected for loss-of-function assays. Following transfection with the shOGFRP1 plasmid, the effectiveness of knockdown was confirmed through qRT-PCR analysis (Fig. 11d) before proceeding with further experiments involving these cells.

### Function and Mechanism of OGFRP1 in EC

**Fig. 12:**
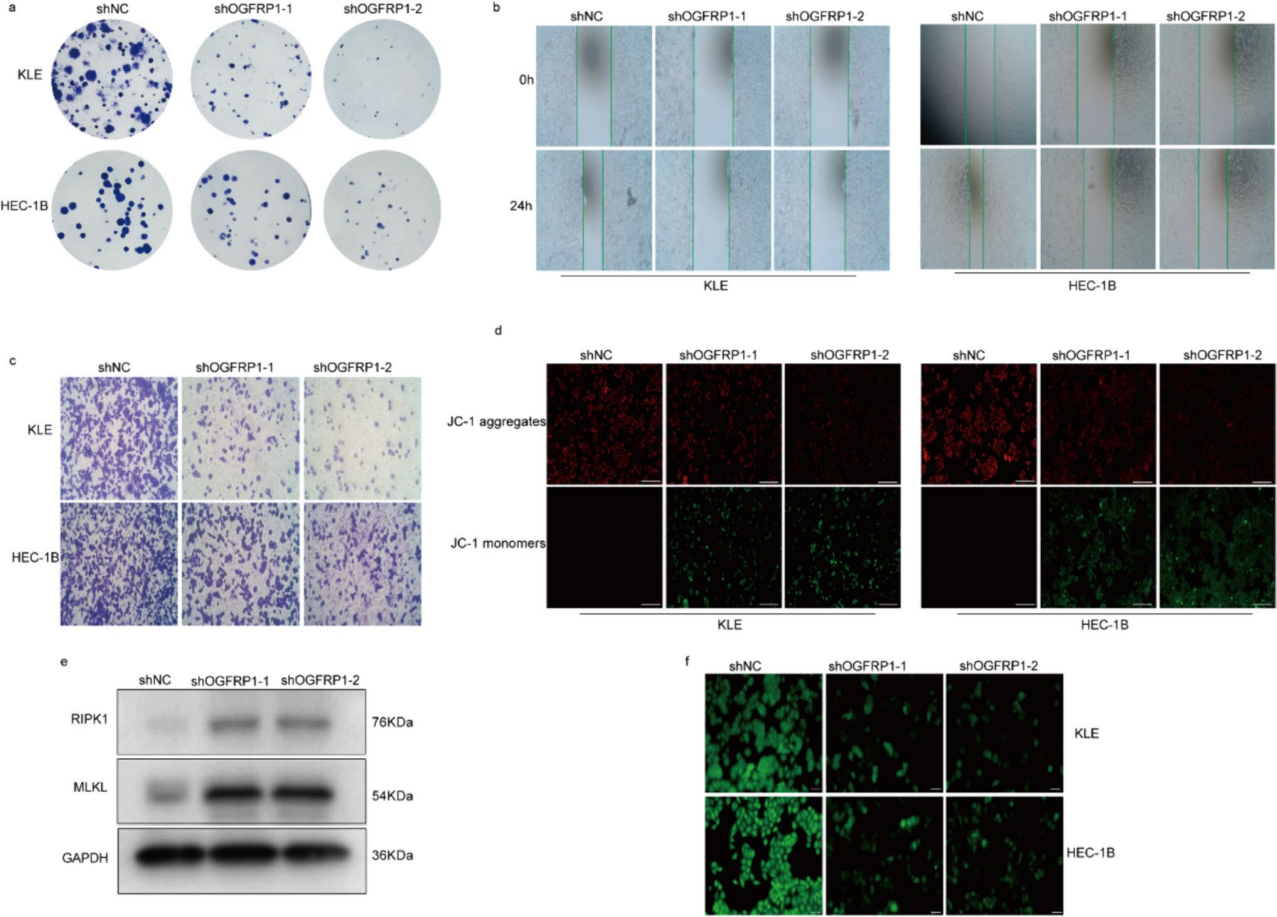
The role of lncRNA OGFRP1 in EC cells. **a**-**c**. Clone formation, wound healing and transwell assay between shNC and shOGFRP1 in KLE and HEC-1B cells. **d**. The change of mitochondrial membrane potential (ΔΨm) in KLE and HEC-1B cells. **e**. The protein levels of RIPK1 and MLKL. GAPDH is an internal parameter. **f**. MPTP opening detection in KLE and HEC-1B cells

To gain a deeper understanding of the impact of OGFRP1 on the malignant progression of EC, we assessed the proliferation and migration capabilities of EC cells. Cloney formation assay indicated that inhibition of OGFRP1 significantly impaired the ability of KLE and HEC-1B cells to form colonies, thereby diminishing cell proliferation (Fig. 12a). The transwell and wound healing assays demonstrated a significant reduction in the migratory ability of EC cells following the inhibition of OGFRP1 (Fig. 12b and c). These results indicated that OGFRP1 may has a significant impact on the development of EC. In addition, to identify the effect of OGFRP1 on mitochondrial function, we examined mitochondrial membrane potential (ΔΨm) and mitochondrial permeability transition pore (MPTP), which are key events that cause cell death. With OGFRP1 knockdown, the ΔΨm detected by JC-1 probe were both decreased in KLE and HEC-1B cells (Fig. 12d). Moreover, intracellular mitochondrial Calcein green fluorescence was diminished after OGFRP1 silencing, which proved that MPTP was in an open state, and the membrane permeability of mitochondria was altered, which eventually led to apoptosis (Fig. 12f). The protein levels of RIPK1 and MLKL, key indicators of cell necrotic apoptosis, were both up regulated in shOGFRP1 treated group (Fig. 12e). Overall, our results partially suggested that OGFRP1 may cause necroptosis of EC cells by disrupting the membrane permeability of mitochondria.

**Fig. 13:**
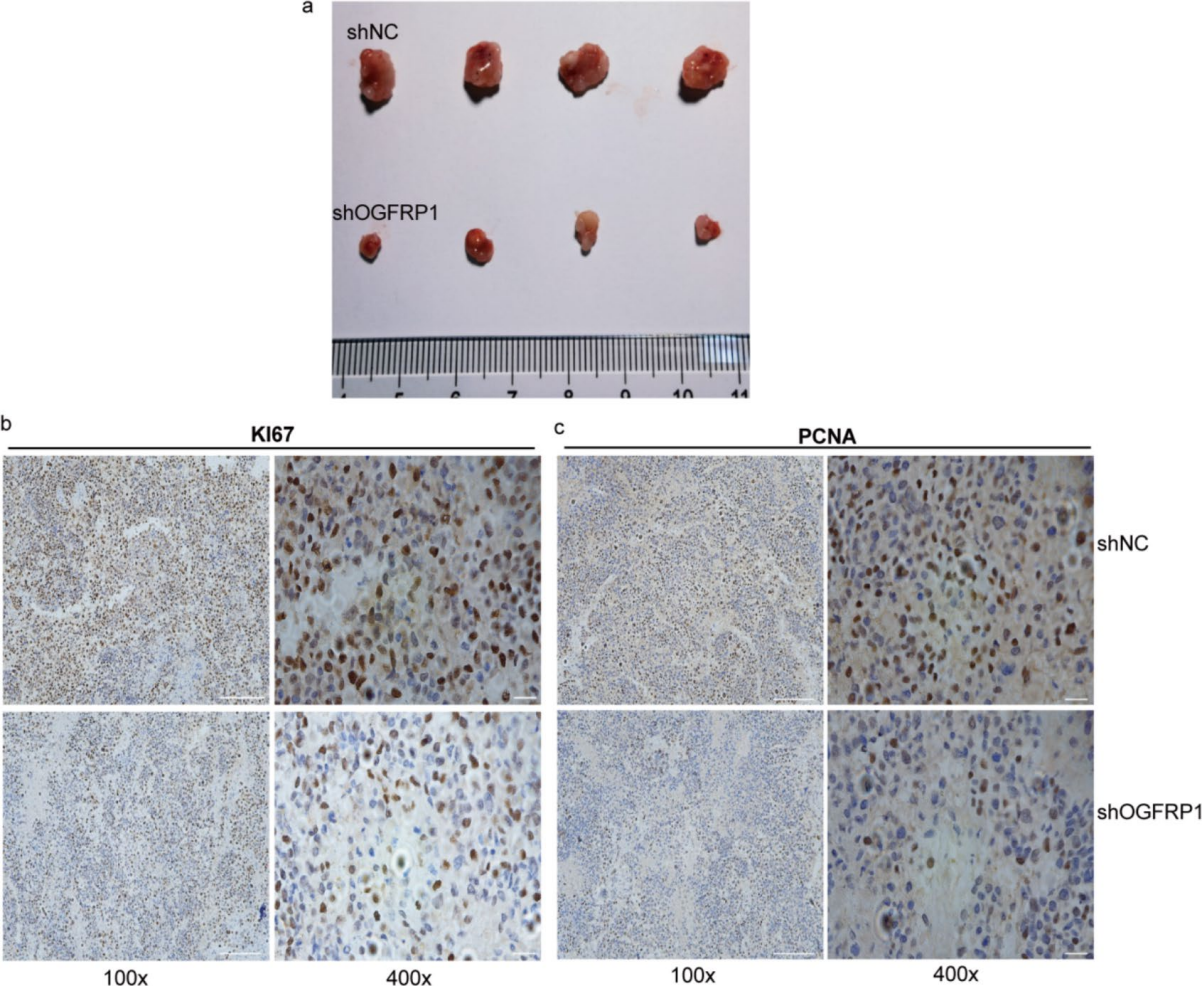
LncRNA OGFRP1 enhance tumor growth in living organisms. **a**. The tumor xenograft volume in nude mice when injected with HEC-1B cells transfected with shOGFRP1 compared to shNC. **b**-**c**. The differential expression of KI67 and PCNA in tumor tumor xenograft between shNC and shOGFRP1 by immunohistochemistry (IHC)

To assess the effect of OGFRP1 on tumor proliferation, a nude mouse tumorigenicity assay was performed. At 35 days after naïve mouse injection with a shOGFRP1 plasmid stably transfected into HEC-1B cells, tumor volumes were significantly reduced (Fig. 13a). Furthermore, the IHC results revealed that the quantity of KI67-positive cells present in the tumor tissues of nude mice with shOGFRP1 was significantly lower in comparison to those treated with shNC. Additionally, the OGFRP1 inhibition group exhibited decreased expression of PCNA in tumor tissues. These data suggested that OGFRP1 may play a crucial role on development of EC tumors (Fig. 13b–c).

## Discussion

Based on bioinformatics analysis, nine prognostic MRLs were identified. These MRLs predicted OS with a high degree of accuracy and specificity. Our study confirmed that nine MRLs exhibit a strong correlation with TMB, TME, and chemotherapy sensitivity in EC. Although several treatments have been used clinically in patients with EC and have shown curative effects, patients with advanced-stage tumors, recurrent EC, or serious histology have poor outcomes [14]. Hence, identification of innovative prognostic biomarkers that can effectively forecast the outcome of EC patients and guide clinical decision-making was essential. Previous studies had identified necroptosis-related signatures as potential prognostic biomarkers and treatment targets in EC [15]. Further study similarly revealed that the prognostic signature of Carcinogenesis-related genes could potentially function as a predictor of anticancer immunotherapy response in EC [16].

In 1979, Hunter and Haworth produced melphalan, prednisolone, and thalidomide (MPT) for the first time [17]. MPT had been reported to induce oxidative phosphorylation uncoupling and activate the mitochondrial apoptosis pathway mediated by CypD [18]. GSK3B resides at the nexus of multiple signaling pathways implicated in regulation of MPT, which causes mitochondrial clearance via mitophagy [19]. Galluzzi identified MPT-driven necrosis as an innovative programmed mortality process [8]. Numerous studies had linked MPT-induced necrosis to acute kidney damage and cardiac injury, as well as to prognosis associated with numerous tumors, including hepatocellular carcinoma, melanoma, and hepatocyte pyroptosis [20–24]. Recently, Zhuang found that MnS nanocapsules may interfere with the permeability transition pore status and facilitate MPT, thereby inducing cell apoptosis for tumor treatment, thereby indicating that MPT may be a novel therapeutic agent for cancer [25]. Therefore, it is essential to better grasp the significance of MPT-driven necrosis in EC. Further studies are necessary to fully comprehend the role of MPT-induced necrosis in EC.

LncRNAs function as oncogenes, tumor suppressors, and novel biomarkers in a broad range of cancers because of their tissue-specific expression patterns across the genome. However, whether MRLs have prognostic value in patients with EC remains unclear. Therefore, we developed a risk model constructed by nine MRLs that might function as an indicator guiding for prognostic prediction in EC. Among the nine MRLs identified, LINC00996 is a novel tumor suppressor that reduces tumorigenesis and metastasis of lung adenocarcinoma via signaling pathways, such as JAK-STAT3 and cell adhesion molecules, similar to antigen processing and presentation [26]. Our results also indicated that LINC00996 may be a protective indicator in patients with EC. OGFRP1, owing the highest coefficient, was highly expressed in patients with high-risk scores and showed infaust prognostic outcomes in EC. In the existing research, OGFRP1 was investigated to enhance the growth of gastric cancer cells and inhibit cell death and sensitivity to radiation by controlling the miR-149-5p/MAP3K3 pathway [27], which has been demonstrated to play a similar role in choriocarcinoma through the miR-4731-5p/HIF3A axis [28]. According to our results, OGFRP1 was up-regulated in EC cells and substantially promote migration and proliferation. These functions might be exerted by influencing the changes of mitochondrial membrane permeability in EC cells, which ultimately affected cell survival. In addition, silencing of OGFRP1 inhibited the growth of subcutaneously transplanted tumors in nude mice and decreased the malignancy of tumors. However, the main limitation of this study was that the mechanism of upstream and downstream regulation of OGFRP1 in mitochondrial function has not been explored, which need further researches.

In addition, GO analysis showed that the DEGs were predominantly associated with remodeling of the extracellular matrix, which promotes the invasion and proliferation of certain cancer cells [29]. Malignancies frequently displayed a dense ECM that substantially impairs drug penetration, restricts therapeutic efficacy, and promotes metastasis. Furthermore, the findings from the GSEA analysis revealed that the high-risk group exhibited heightened activity in pathways associated with tumor development, including the cell cycle, DNA mismatch repair (MMR), and Hedgehog (Hh) signaling pathways. MMR status is an indispensable shunt node in the molecular typing of EC and plays an irreplaceable role in the evaluation of patient prognosis, genetic screening, and curative effect prediction [30]. Normally, the Hh signaling pathway in adults was almost completely silenced in tissues, and abnormal activation of this pathway can lead to cancer [31]. Cancer stem cells maintain stemness by regulating pluripotency genes, including Nanog, Sox2, and Bmi1, in response to Hh ligands secreted by neighboring stromal cells [32]. In addition, we discovered that inflammatory factor-related pathways, such as “chemokine signaling pathway” and “cytokine-cytokine receptor interaction”, are more active in low-risk group compared to high-risk group. In the TME, cytokines are important cell communication mediators, and some cytokines contribute to the host's antitumor response. However, cancer is characterized by dysregulated cytokine production and function [33]. The mentioned pathways above, which may be linked to the cellular specifying process and ultimately organ growth, may represent innovative therapeutic and preventative targets for EC.

TME plays key role in immune-suppression or activation, and has important reference significance for tumor prevention and immunotherapy [34]. Functionally, MRLs may promote sensitivity regulation of immune responses by modulating immune patterns and TMB. Das et al. [35] found that DNM3OS over-expression promotes phagocytosis, immune gene expression, and an inflammatory phenotype in macrophages. Furthermore, Zhou revealed that OGFRP1 was involved in shaping the profile of tumor-infiltrating B lymphocytes in bladder cancer, indicating its potential significance for prognosis and immunotherapy. These findings suggest that OGFRP1 may play a role in the restructuring of TME [36].

To examine the relationship between immunological competence and risk scores, we conducted a comparative analysis of immune cell infiltration and immune-related pathways between high-risk and low-risk groups. Our findings revealed that the infiltration levels of most immune cells were significantly lower, and the activity of various immune-related pathways was notably reduced in the high-risk group. This pattern suggests the presence of a “cold immunity” state in EC. Specifically, in the high-risk cohort, key immune-related functional indicators—including cytolytic activity, human leukocyte antigen (HLA), T cell co-stimulation, type II interferon (IFN) response, T cell co-inhibition, chemokine receptor (CCR), tumor-infiltrating lymphocytes (TILs), and CD8 + T cells—demonstrated diminished activity. The activity of type I IFN response and ADCs was higher. TILs abundance had been shown to associated with a biomarker associated with favorable prognosis for many solid tumors [37]. Type II IFN respnose is a key element of antiviral immunity, which also thought to be one of the causes of immune escape [38]. However, Type I IFN response not only promote the infinite proliferation of cancer cells but also cause cancer exosomes to release highly expressed levels of immune checkpoint receptor ligand properties [39]. These results indicate the significant role of IFN response in EC. It has been reported that cytolytic activity is associated with antiregulatory activity that limits the immune response [40]. T-cell co-inhibition pathway interference effectively enhances protective immunity in tumor formation and pathogen persistence [41]. Furthermore, patients classified in the low-risk group exhibited greater CCR activity compared to those in the high-risk group. This observation may imply that individuals classified within the low-risk category are more inclined to exhibit a positive response to inhibitor therapy, which could in turn lead to enhanced outcomes.

The responses of tumor patients to immunotherapy can be evaluated by TMB, which was used to reflect the number of somatic mutations in a tumor and are often considered biomarkers in immunotherapy [42]. Patients with TMB-High may be more readily recognized by immune cells, thereby increasing their potential to benefit from immunotherapy. Our findings indicated that TMB is more prevalent within the low-risk group. Survival curves that integrate TMB data with risk stratification reveal that individuals exhibiting both TMB-High and low-risk scores experience the most favorable prognosis. TIDE was employed to anticipate immune escape in patients with EC undergoing immunotherapy. As the TIDE score escalates, the likelihood of tumor immune escape during immunotherapy correspondingly increases. Our investigation revealed that patients classified within the high-risk group exhibit elevated TIDE scores, potentially indicating a poorer response to immunotherapeutic interventions. Consequently, it is imperative that we direct greater attention to immunotherapy strategies for high-risk patients.

Furthermore, four prospective medications were evaluated, CMK, midostaurin, dasatinib and paclitaxel, all of which exhibited reduced IC50 values in a high-risk cohort. Conversely, patients classified as low risk exhibited greater sensitivity to axitinib, docetaxel, metformin and temsirolimus. Recently, dasatinib has shown encouraging clinical efficacy and tolerable toxicity when administered in combination with paclitaxel and carboplatin in clinical trials, particularly in cases of recurrent EC [43]. The remaining two agents with low IC50 values have not yet been described for EC. The findings of our study indicate that midostaurin and CMK have the potential to serve as EC therapeutic agents to enhance clinical effectiveness against EC.

In conclusion, we identified an MRLs signature as a novel prognostic biomarker and potential target for therapy in patients with EC. Furthermore, this prognostic signature will provide valuable insights for EC diagnosis.

## Materials and Methods

### Data Collection

Data of RNA-seq, mutation data and clinical data were derived from the TCGA database (https://portal.gdc.cancer.gov/). The information on MRGs was sourced from prior research (see Supplementary S5).

### Discrimination of MRLs in EC

Pearson correlation analysis was utilized to identify potential MRLs by assessing the expression levels of MRGs and lncRNAs (*P* < 0.001, | | R |> 0.4), and a Sankey diagram was generated to visualize their co-expression relationship. Following this, the “limma” package was employed to identify significantly different MRLs (*P* < 0.05).

### Establishment and Verification of the MRLs

The samples were randomly divided into equal training and testing cohorts at a 1:1 ratio. The training set was utilized to develop prognostic risk scores (MRLs), while the testing set was reserved for validation purposes. Prognostic MRLs were identified through a univariate Cox regression analysis. Subsequently, a LASSO analysis was conducted on the significant prognostic lncRNAs, with parameter estimates determined via tenfold cross-validation. The final selection of optimal prognostic MRLs (*P* < 0.05) was accomplished through multi-Cox regression analysis, leading to the construction of the risk model. The associated risk score was then computed using the following model,

$$\text{RiskScore }=\text{ n }\sum \text{ i}=1\text{ Coefi }*\text{ xi}$$

The samples were separated into high- and low-risk groups based on the median risk score. The OS of EC patients in each group was determined using a Chi-square test. ROC curve and C-index was calculated to evaluate the precision of model.

### The Construction and Validation of the Prognosis Model

Uni- and multi-variate Cox regression analyses were used to evaluate the individual prognosis impact of the risk score and important clinical characteristics. Subsequently, nomograms were constructed using packages like “rms”, “survival” and “regplot” to predict patients' survival probabilities at 1, 3 and 5 years. Calibration curves were generated to illustrate the disparities between the anticipated and observed results of the nomogram.

### Perform PCA and Functional Enrichment Analysis

PCA was constructed using “Scatterplot3D” and “limma” and package to investigate how patients with different risk scores are distributed. GO analysis was carried out utilizing the “ggpubr”, “ggplot2”, “colorspace”, “circlize” and “stringi”, packages to illustrate the DEGs in high- and low-risk groups. Additionally, GSEA analysis was performed employing the “enrichplot” and “clusterProfiler” packages to further characterize the enriched pathways in distinct datasets.

### TMB and TIDE Analysis

The “maftools” package was employed for analyzing the variations in TMB between the high- and low-risk groups. The Survival package was utilized to assess the relationship among patient survival and TMB. The scoring file for TIDE was obtained from the website (http://tide.dfci.harvard.edu).

### Examination of TME and Immune Checkpoint

The association between different risk score values and immune cell subpopulations was evaluated using spearman correlation analysis, which included the CIBERSORT, TIMER, XCELL, QUANTISEQ, MCP counter, EPIC and CIBERSORT tools. The Wilcoxon signed-rank test was conducted to assess for significant differences, with a significance level of *p* < 0.05. Subsequently, the “limma” and “GSVA” packages were utilized to explore variations in immune-related functions among endometrial cancer patients. Lastly, the comparison of immune checkpoints activation between the two groups was carried out using the “ggpubr” package.

### Investigation of Models in Clinical Therapy

The “Prophet”, “ggplot2”, and “ggpubr” packages were utilized to predict the IC50 of EC. This aimed to identify potential therapeutic drugs that align with the model and could be considered for treating endometrial cancer.

### Single-Cell Sequencing Data Analysis

The NCBI SRA database contained the single-cell sequencing data SRP349751 that included five primary tissues from endometrioid endometrial carcinoma (EEC), and five normal endometrial tissues. Low-quality cells were eliminated from the expression matrix using the "Seurat" R package, and the "NormalizeData" package was used to integrate and normalize the data. Following principal component analysis (PCA) using "RunPCA," cells were alternatingly clustered at the maximum resolution using "FindClusters." The data was shown using the t-distributed stochastic neighborhood embedding method (tSNE).

### qRT-PCR and Western Blot

RNA extraction, qRT-PCR and western blotting were all carried out as described previously [44]. Primers employed are showed in Supplementary S6.

### Cell Lines Culture

The EC cell lines utilized in this study, specifically HEC-1-A, HEC-1-B, KLE, and Ishikawa, were obtained from the ATCC. Normal endometrial cells were isolated from healthy uterine tissue. The KLE, HEC-1-B, Ishikawa, and normal endometrial cells were cultured in DMEM/F12 medium supplemented with 10% FBS (Gibco), whereas HEC-1-A cells were maintained in McCoy's 5A medium also containing 10% FBS. All cell lines were incubated in a controlled environment with 5% CO2 at 37 °C, and both penicillin and streptomycin were added at a concentration of 100 µg/mL to prevent bacterial contamination.

### Cell Transfection

The small hairpin RNAs (shRNAs) targeting OGFRP1 were synthesized and subsequently cloned into the PGMLV-hU6-MCS-CMV-Puro-WPRE vector by Genomeditech (Shanghai, China). The specific shRNA sequences utilized in this study are provided in Supplementary S6. To create stable cell lines with OGFRP1 knockdown, we employed a lentiviral-mediated delivery system at a multiplicity of infection (MOI) of 30, coupled with the use of polybrene. Following infection, cells were selected with 1.5 g/mL of puromycin for 48 h in order to establish stable cell lines. A scramble shRNA was utilized as a control in these experiments. For plasmid transfection, KLE and HEC-1B cells were cultured in six-well plates for a duration of 24 h prior to the transfection with 4 µg of expression plasmid. The transfection was carried out using Lipo3000 (Invitrogen, CA, USA), following the manufacturer’s instructions. Total RNA was harvested 48 h post-transfection to assess transfection efficiency via qPCR.

### Colony Formation Assay

Cells that had been transfected were placed in a 6-well plate a nd kept in an incubator with complete medium at 37°C for 14 days. Following this, the cells were treated with 4% paraformaldehyde for fixation and stained with 2% crystal violet.

### Transwell Assay

KLE and HEC-1B cells were suspended at a concentration of 1 × 10^4^ in serum-free DMEM-F12 and placed into MERCK chambers with an 8 mm diameter. A complete medium was added to a deep-well 24-well plate containing transwell chambers. Following incubation, cells on the upper membrane surface were gently removed using a cotton swab. Subsequently, staining with crystal violet was performed, and five representative microscopic fields were randomly chosen to quantify the cells and measure the migration rate.

### Wound Healing Assay

Cells were seeded in six-well plates and incubated for 24 h until reaching complete confluence. Cell injuries were inflicted using a 10ul pipette tip. Subsequently, the cells were maintained in a serum-free medium, and the wound width was documented at 0h and 24h using microscope.

### Measurement of Mitochondrial Membrane Potential (ΔΨm)

KLE and HEC-1B cell lines were seeded into 96-well plates at a density of 1 × 10^4 cells per well and subsequently transfected with shOGFRP1. Following the manufacturer’s instructions, the mitochondrial membrane potential was assessed using a JC-1 Detection Kit (C2006, Beyotime). Fluorescent images were acquired using an inverted fluorescent microscope.

### MPTP Opening Detection

The MPTP was detected with MPTP detection kit following the manufacturer's instructions (KTA4002, Abbkin). Images were captured using an inverted fluorescent microscope.

### Tumor Xenograft Model

The Animal Care Committee of Tongji Medical College has provided approval for all animal experiments conducted in this study. Four-week-old female BALB/c-nu nude mice were acquired from Vital River (Beijing, China) and housed in a specific pathogen-free environment. HEC-1B cells, transfected with shOGFRP1 and the vehicle control, were washed with serum-free medium prior to being subcutaneously injected into the nude mice at a dosage of 6 × 10^6 cells per injection site. After 35 days post-implantation, the mice were euthanized, and the tumors were carefully excised for subsequent analysis.

### Statistics

The laboratory results are presented as the mean ± SEM. Comparisons between group means were performed using Student's t-test and ANOVA, as detailed in the figure captions. The statistical analyses were conducted using GraphPad Prism version 8.1.2 software.

## Supplementary Information

Below is the link to the electronic supplementary material.

Supplementary file1 (XLSX 57447 KB)

Supplementary file2 (XLSX 30 KB)

Supplementary file3 (XLSX 43 KB)

Supplementary file4 (XLSX 17 KB)

Supplementary file5 (XLSX 10 KB)

Supplementary file6 (XLSX 10 KB)

Supplementary file7 (XLSX 27 KB)

Supplementary file8 (XLS 1 KB)

Supplementary file9 (XLTX 12 KB)

Supplementary file10 (XLS 144 KB)

## Data Availability

The data that support the findings of this study are openly available in TCGA at https://tcga.xenahubs.net.
